# Comparison of HCG Trigger versus Dual Trigger in Improving Pregnancy Outcomes in Patients with Different Ovarian Responses: A Retrospective Study

**DOI:** 10.1155/2024/2507026

**Published:** 2024-05-31

**Authors:** Ke Xu, Jinrong Wang, Shuangshuang Yang, Zhenjing Wang, Ning Hou, Mei Sun

**Affiliations:** ^1^Center for Reproductive Medicine, Shandong University, Jinan 250012, Shandong, China; ^2^Key Laboratory of Reproductive Endocrinology of Ministry of Education, Shandong University, Jinan 250012, Shandong, China; ^3^Shandong Provincial Hospital Affiliated to Shandong First Medical University, Jinan, Shandong, 250012, China; ^4^National Research Center for Assisted Reproductive Technology and Reproductive Genetics, Shandong University, Jinan 250012, Shandong, China; ^5^Department of Pharmacology, Institute of Materia Medica, Chinese Academy of Medical Sciences and Peking Union Medical College, Beijing 100050, China; ^6^Shandong First Medical University (Shandong Academy of Medical Sciences), Jinan 250117, Shandong, China

## Abstract

**Objective:**

During in vitro fertilization-embryo transfer (IVF-ET) treatment, the reproductive endocrine regulatory mechanisms hold pivotal importance. Specifically, the serum estradiol (*E*_2_) level during ovulation emerges as a critical factor influencing pregnancy outcomes. This retrospective study aimed to comprehensively compare two common clinical regimens based on the grouping of serum *E*_2_ levels and the number of oocytes retrieved on the trigger day. Our objective was to evaluate the pregnancy outcomes in IVF-ET patients across different ovarian response groups, exploring the efficacy of the dual-trigger and single-trigger regimens to provide valuable insights for optimizing clinical strategies in the context of IVF-ET.

**Methods:**

A retrospective analysis was conducted on the clinical data of 2778 infertile patients who underwent ART (IVF/ICSI). Subsequently, a detailed statistical analysis was performed on 1032 patients following an antagonist regimen. Participants were categorized into single-trigger and dual-trigger groups based on real-world trigger protocols, considering different ovarian responses. Comprehensive statistical assessments were conducted on baseline characteristics, ovulation induction, and pregnancy outcomes.

**Results:**

Baseline characteristics and cycle parameters among the three patient groups (high ovarian response, normal response, and poor response) exhibited no significant differences between the dual-trigger and single-trigger regimen groups. Despite the dual-trigger regimen utilizing a significantly lower HCG dose, no notable discrepancies were observed in laboratory results and pregnancy outcomes (embryo transfer rate, pregnancy rate, and live birth rate) for normal and high responders. Remarkably, *E*_2_ levels were higher in the dual-trigger group compared to the single-trigger group. In high and normal responders, the dual-trigger regimen demonstrated increased oocyte counts and oocyte acquisition rates, coupled with decreased transfer cancellation rates attributed to ovarian hyperstimulation syndrome (OHSS). Intriguingly, patients with a poor ovarian response experienced no graft cancellations due to OHSS prevention in either group.

**Conclusion:**

For patients with high and normal ovarian responses, the utilization of a dual-trigger regimen on the trigger day effectively mitigates the risk of OHSS. Our large sample study supports the substitutability of the dual-trigger regimen over the single-trigger regimen without compromising pregnancy outcomes. However, this conclusion is not applicable to patients with poor ovarian responses. The results of this study highlight the necessity of adopting a customized and individualized treatment approach that should be based on the patient's ovarian response. Additionally, recognizing the pivotal role of the endocrine environment in influencing pregnancy outcomes and the occurrence of OHSS, further exploration of the effects of different triggering regimens on endocrine parameters is warranted. Such investigations will contribute to enhancing the reproductive outcomes of IVF-ET technology.

## 1. Introduction

Infertility affects approximately 48 million couples and 186 million individuals globally, underscoring the significance of assisted reproductive technology (ART) as a pivotal treatment modality [1]. Controlled ovarian hyperstimulation (COH) plays a crucial role in ART procedures such as in vitro fertilization and embryo transfer (IVF-ET) and intracytoplasmic sperm injection (ICSI), aiming to achieve a higher yield of oocytes.

Ovulation is intricately linked to the integrity of the hypothalamic-pituitary-gonadal (HPG) axis, and ovulation triggering stands out as a pivotal step in COH. Typically, human chorionic gonadotropin (HCG) and gonadotropin-releasing hormone agonist (GnRHa) serve as triggers, closely mimicking physiological luteinizing hormone (LH) peaks and inducing ovulation approximately 36–40 hours later. However, the extended half-life and increased receptor affinity of HCG over LH elevate the risk of OHSS, an iatrogenic complication [2]. OHSS often occurs in the luteal phase after ovulation induction or early pregnancy, potentially causing respiratory distress and gastrointestinal issues and endangering the lives of patients in severe cases. To mitigate OHSS risk, GnRHa is increasingly favored as a trigger, as it induces endogenous LH peaks more closely resembling natural hormonal patterns [3]. Studies indicate that GnRHa triggers can reduce vascular endothelial growth factor mRNA expression, subsequently lowering OHSS incidence [4]. For patients with a high ovarian response and the associated risk of OHSS, selecting GnRHa as the trigger has demonstrated efficacy in OHSS prevention. In antagonist cycles, GnRHa triggers are preferred due to heightened pituitary sensitivity.

The GnRH antagonist protocol, gaining widespread application, proves effective for patients with varying ovarian responses, offering positive clinical outcomes [5]. Despite concerns about reduced corpus luteum function and fresh-cycle embryo transfer rates, frozen embryo transfer presents a viable alternative. Approximately 5.2% of infertility patients exhibit insensitivity to single GnRHa triggers, often observed in those at high risk of OHSS [6].

The emergence of the dual-trigger regimen, combining HCG and GnRHa, represents a recent development with limited real-world clinical data. Selection of the trigger protocol is guided by serum estradiol (*E*_2_) levels, follicle count, patient parameters (height and weight), and previous ovulation responses. In high-response populations, where follicle counts exceed 20 or oocytes obtained surpass 15, the dual trigger is increasingly recommended [7]. Compared to the GnRHa single trigger, the dual trigger elevates endogenous LH and FSH levels, enhances oocyte maturity, and increases retrieval rates without compromising success rates. Notably, dual triggering reduces OHSS risk and enhances pregnancy outcomes compared to HCG single triggering [8].

This study reviews the clinical data of trigger protocols in patients with different ovarian responses to the antagonist protocol, using propensity score matching (PSM) to control for confounding factors and evaluating the efficacy and safety of the dual trigger, combining GnRHa with low-dose HCG. The observed pregnancy outcomes provide valuable evidence for optimizing clinical strategies in diverse responder populations. The impact of dual triggers on pregnancy outcomes in different response populations.

## 2. Materials and Methods

### 2.1. Study Subjects

This observational retrospective study was conducted in a single regional hospital in China, and approval was obtained from the Ethics Committee of Reproductive Hospital Affiliated to Shandong University on October 24, 2022. The ethical approval number is 2022103. The data extraction was performed in the hospital HIS system. This study retrospectively analyzed 2778 infertile women who underwent in vitro fertilization/intracytoplasmic sperm injection (IVF/ICSI) fertility treatment from January 1, 2018, to December 31, 2020, at the Center for Reproductive Medicine of Shandong University. All enrolled patients signed an informed consent form to allow data collection for research purposes without violating patient privacy or ethical norms. The quality of sperm is qualified by routine semen examination [9].

Inclusion criteria are as follows: (1) IVF/ICSI patients with the GnRH antagonist protocol; (2) aged 20–40 years; (3) cycle number ≤2; and (4) with a body mass index (BMI) of 18–30 kg/m^2^.

Exclusion criteria are as follows: (1) suffering from an endocrine disorder (diabetes mellitus, hyperprolactinemia, thyroid dysfunction, congenital adrenal hyperplasia, cushing syndrome, or polycystic ovary syndrome) and (2) a uterine anomaly confirmed by hysterosalpingography or hysteroscopy.

### 2.2. Regimens for Ovarian Stimulation

All patients are treated with the GnRH antagonist protocol for controlled ovulation induction therapy. Gn is applied to ovulation induction starting at 2 to 3 days of the menstrual cycle, and the amount of Gn is determined according to the patient's age, BMI, basal hormone level, antral follicle count (AFC), etc. Commonly used Gn drugs are Recombinant Follitropin Beta injection (Puregon, N. V. Organon, Netherlands) and Urofollitropin for injection (Lishenbao, Livzon Pharmaceutical Group Inc., China). The monitor follicle grows after applying Gn for 4 days and adjusting the amount of Gn according to the development of follicles. When at least one lead follicle ≥14 mm, the GnRH antagonist Cetrorelix Acetate Powder for injection (Sizekai, Merck Serono Europe Limited, United Kingdom) or Ganirelix injection (ORGALUTRAN, N.V.Organon, Netherlands) acetate 0.25 mg/day is applied to follicle maturation.

When ultrasound detects the presence of two or more follicles ≥18 mm in diameter in both ovaries, a drug trigger is performed that night. Trigger protocols are administered according to the serum estradiol *E*_2_ level and follicular development number on the day of the trigger. Including the HCG alone group (chorionic gonadotrophin for injection, Livzon Pharmaceutical Group Inc., China), the dual trigger protocol HCG was combined with GnRHa (Triptorelin Acetate for injection, Dipherelinel, Ipsen Pharma Biotech, France; Triptorelin, Dabijia, Ferring GmbH, Germany). The dose was adjusted according to hormonal levels and individual characteristics on the trigger day. The retrieval is performed under the guidance of vaginal ultrasound 36 to 38 hours after the trigger.

### 2.3. Grouping

Depending on the ovarian response, patients were divided into three groups: high responders (trigger day *E*_2_ > 4000 pg/ml or >15 oocytes obtained), normal responders (trigger day *E*_2_ ≤ 4000 and 4 ≤ number of oocytes obtained ≤15), and poor responders (number of oocytes obtained ≤3) [10]. In real-world clinical practice, based on experience and patient assessment, physicians usually use the dual trigger group with GnRHa and HCG in high-response patients who have OHSS risk, and those without risk use a single trigger with HCG.

### 2.4. Outcome Variables

1. Characteristics of patients at baseline: age, BMI, infertility type, causes of infertility, baseline follicle stimulating hormone (FSH), baseline LH, baseline estradiol (*E*_2_), antimullerian hormone (AMH), baseline AFC.
2. Characteristics of ovarian stimulation: total dose of Gn, duration of stimulation, number of large follicles above 14 mm in diameter on the trigger day, serum LH on the trigger day, serum *E*_2_ on the trigger day, serum P on the trigger day, endometrial thickness on the trigger day, the dose of HCG, the number of oocytes, the oocyte retrieval rate, the number of fertilizations, the fertilization rate, the number of high-quality embryos, and the high-quality embryo rate.
3. Pregnancy outcome indicators: the embryo transfer rate, clinical pregnancy rate, biochemical pregnancy rate, live birth rate, ectopic pregnancy rate, abortion rate, transplant cancellation rate (reasons: prevention of OHSS, high *P* value, endometrial factor, embryonic factor, oocyte factor, and others), and OHSS incidence rate.

### 2.5. Statistical Analyses

PSM is a statistical method used to process data. In observational studies, due to various reasons, there are more biases and confounding variables in the data. The PSM is precisely designed to reduce the impact of these biases and confounding variables in order to make a more reasonable comparison between the experimental group and the control group. In assisted reproduction, the patient's baseline conditions, ovarian response, and trigger-day ovarian indicators can all affect pregnancy outcomes. Therefore, whether to use a dual trigger as the dependent variable or exposure factors (patient baseline characteristics, ovarian function, stimulus indicators, etc.) that affect pregnancy outcomes as independent variables, a binary logit model is constructed for 1 : 1 nonreplacement nearest neighbor matching (NNM) of PSM.

Using SPSS 23.0, the continuity variable is first normalized, and the large sample (*n* > 50) is tested using the Kolmogorov–Smirnov test. The indicators that meet the normality test are analyzed by the Student's *t*-test, and the measurement data are presented as mean ± standard deviation (*X* ± *S*). The indicators that did not meet the normality test use the Mann–Whitney *U*-test when classified into two groups and the Kruskal–Wallis test when more than two groups are presented by the median *M* and quartile spacing (P25 and P75). For intergroup comparison of categorical variables, the sample size *n* ≥ 40 and the theoretical frequency *T* ≥ 5 use Pearson's chi-squared test, *n* ≥ 40 and 1 ≤ *T* < 5 use the likelihood-ratio test, and *n* < 40 and *T* < 1 use the Fisher chi-square test, presented as frequencies and percentages. *P* < 0.05 was statistically significant for the difference.

## 3. Results

**Figure 1:**
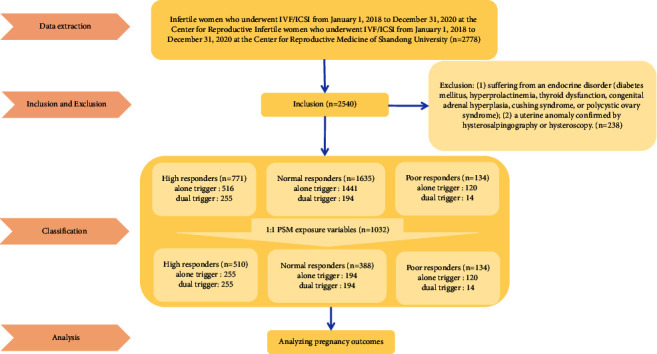
Flowchart of infertile patients who underwent IVF/ICSI.

A total of 2540 patients met the inclusion criteria. There were matching confounding factors (age, BMI, infertility type, causes of infertility, baseline FSH, baseline LH, baseline *E*_2_, AMH, total dose of Gn, duration of stimulation, number of large follicles above 14 mm in diameter on the trigger day, serum LH on the trigger day, serum *E*_2_ on the trigger day, serum P on the trigger day, and endometrial thickness on the trigger day) between high responders and normal responders. The final analysis consisted of a total of 1032 patients, including 510 high responders, 388 normal responders, and 134 poor responders (Figure 1). The three groups before and after matching were compared in terms of baseline characteristics, ovarian stimulation characteristics, and pregnancy outcomes in two trigger groups.

Table 1 shows a comparison of the characteristics of patients in the dual trigger group and the HCG alone group before and after matching against patients at baseline (age, BMI, infertility type, causes of infertility, FSH, LH, *E*_2_, AMH, and AFC). The results showed statistically significant differences before the propensity score matched the two triggering regimens before matching with the infertility type and AFC in high responders and age, FSH, LH, and AFC in normal responders (*p* < 0.01). However, there were no significant differences in baseline and cycle stimulation data between the different responding populations applying the two triggering regimens after the propensity score matched (*P* > 0.05).

Table 2 shows statistically significant differences in serum *E*_2_, HCG dose, and the number of large follicles in the three responding patient populations with the dual trigger regimen (*P* < 0.01), and no statistically significant differences were found in the rest of the indices. After the propensity score was matched between the populations of the two regimens, there were significant differences in the two trigger groups and HCG doses with *p* value ≤0.001 in different responders, and the rest of the results were not statistically different. However, most laboratory results in the three ovarian response groups showed slightly higher values in the dual trigger group than in the HCG alone group, proving that there is still an advantage in the dual trigger group.

In the retrieval of patient data, the usual dose of HCG for the single-trigger group is 6000IU, 8000IU, or 10000IU; the usual dose of HCG for the dual-trigger group is 2000IU or 4000IU, and the specific dosage needs to be adjusted according to the patient's laboratory indicators. We can find that in order to prevent adverse reactions to HCG, the dosage of HCG has been reduced for the high responders.

Table 3 shows a comparison of patient characteristics in pregnancy outcomes after matching in the dual trigger group and HCG alone group. There were no significant differences in pregnancy outcomes in the different trigger groups of normal and high responders as displayed in the results (*P* > 0.05). Moreover, although there was no statistically significant difference between the two triggering regimens in high responders, the incidence of OHSS was lower with dual triggering (3.92 vs. 1.96%). In poor responders, the embryo transfer rate of the HCG-alone trigger group is significantly higher than that of the dual trigger group, and there are no cases of transfer cancellation for OHSS prevention in both groups.

## 4. Discussion

*E*_2_ is a hormone secreted by ovarian follicular cells that plays an important role in maintaining the growth and development of oocytes. Previous studies have shown an increased risk of OHSS in patients with *E*_2_ > 3500 pg/ml [11]. A very high *E*_2_ level and too many oocytes retrieved increase the incidence of moderate-to-severe OHSS [12]. Therefore, the risk of OHSS can be predicted clinically by assessing the trigger-day *E*_2_ level and the number of oocytes retrieved. In this study, the *E*_2_ level of the dual trigger group was higher than that of the single trigger group in the high-response patients and the normal-response patients.

OHSS remains a prevalent complication of COH during ART. Reducing the dose of gonadotropins is the most effective way to prevent OHSS. Recently, the use of Myo-Inositol oral supplementation during ovarian stimulation has been found to reduce the amount of gonadotropins and the time of ovarian stimulation [13]. The combination of GnRHa with an HCG dual trigger can also reduce the dose of HCG, which has shown promising results in preventing OHSS, as previously demonstrated; however, its definitive role necessitates further substantiation [14]. The theoretical underpinning for the dual trigger, encompassing GnRHa and HCG, lies in its potential to maintain stable luteal function and enhance pregnancy outcomes. Previous research studies, including those conducted by Şükür and Albeitawi, support the superiority of the dual trigger in normal responders [2, 15]. Chung et al. endorse the dual trigger protocol as an effective strategy for in vitro fertilization in high responders without compromising fresh cycle pregnancy outcomes [16]. Li et al.'s study highlights the dual trigger's capability to prevent severe OHSS while maintaining an excellent high-quality embryo rate in high-ovarian responders following GnRH antagonist protocols [17]. Our current study aligns with these findings, revealing a lower transplant cancellation rate in both high and normal responders within the dual trigger group compared to the single trigger group, attesting to the risk reduction in OHSS occurrence. Importantly, there were no significant differences observed in pregnancy outcomes for high and normal responders, further reinforcing the effectiveness and safety of the GnRHa combined with the low-dose HCG dual trigger protocol when compared to the HCG alone trigger. Contrary to these positive outcomes, Eser A posits that the dual trigger may not yield improved oocyte maturation, clinical pregnancy, or ongoing pregnancy rates in poor responders [18]. Our findings in poor responders corroborate this perspective, with a lower embryo transfer rate observed in the dual trigger group compared to the alone trigger group, signifying potential limitations in its application for this population.

Analyzing the reasons for transplant cycle cancellation, our study underscores the predominant role of OHSS prevention as the primary factor. The absence of a standardized HCG dosage for the dual trigger in clinical practice, often tailored to individual patient conditions and physician experience, may contribute to the observed high transplant cancellation rate [19]. This prompts a critical need for further investigation into optimizing HCG dosage to potentially increase the proportion of fresh embryo transfers without elevating the OHSS risk.

Despite our efforts to match baseline patient characteristics and the positive outcomes observed with the dual trigger, this retrospective analysis has limitations. The inherent biases in clinical preferences for trigger protocols, the smaller population of poor responders, and potential sample loss in matching control baselines are acknowledged. Consequently, deficiencies in embryo transfer rates, biochemical pregnancy rates, clinical pregnancy rates, and other parameters warrant consideration. Larger prospective randomized controlled trials are imperative to comprehensively evaluate whether the dual trigger genuinely improves pregnancy outcomes across diverse ovarian response populations and attains superior clinical results.

## 5. Conclusions

Our findings highlight that in patients with high- and normal-ovarian responses, the utilization of a dual-trigger regimen on the trigger day could mitigate the risk of OHSS. Our large sample study validates the substitutability of the dual-trigger regimen over the single-trigger regimen without compromising pregnancy outcomes, and this strategic approach significantly mitigates the risk of canceling fresh embryo transfers attributed to OHSS prevention while concurrently yielding favorable pregnancy outcomes. However, this conclusion is not applicable to patients with poor ovarian responses. The results of this study highlight the necessity of adopting a customized and individualized treatment approach that should be based on the patient's ovarian response. The expansion of our study's sample size reinforces the robustness of the evidence supporting the dual trigger as a relatively effective and safe regimen. Additionally, recognizing the pivotal role of the endocrine environment in influencing pregnancy outcomes and the occurrence of OHSS, further exploration of the effects of different triggering regimens on endocrine parameters is warranted. Such investigations will contribute to enhancing the reproductive outcomes of IVF-ET technology.

## Figures and Tables

**Table 1:** Comparison between the dual trigger group and the HCG alone group before and after matching: characteristics of patients at baseline.

		High responders						Normal responders						Poor responders		
		Before matching			After matching			Before matching			After matching					
		HCG alone trigger	Dual trigger	*P*	HCG alone trigger	Dual trigger	*P*	HCG alone trigger	Dual trigger	*P*	HCG alone trigger	Dual trigger	*P*	HCG alone trigger	Dual trigger	P
Sample size		*n* = 516	*n* = 255	—	*n* = 255	*n* = 255	—	*n* = 1441	*n* = 194	—	*n* = 194	*n* = 194	—	*n* = 120	*n* = 14	—

Age (years)		30.32 ± 3.79	30.31 ± 3.76	0.973	30.45 ± 3.72	30.31 ± 3.76	0.670	31.12 ± 3.97	31.07 ± 3.53	0.001^*∗∗*^	30.77 ± 3.84	31.07 ± 3.53	0.425	32.54 ± 4.40	33.93 ± 4.58	0.269

Infertility type	Primary	236 (45.74)	142 (55.69)	0.009^*∗∗*^	144 (56.47)	142 (55.69)	0.858	701 (48.65)	95 (48.97)	0.933	100 (51.55)	95 (48.97)	0.612	46 (38.33)	8 (57.14)	0.175
	Secondary	280 (54.26)	113 (44.31)		111 (43.53)	113 (44.31)		740 (51.35)	99 (51.03)		94 (48.45)	99 (51.03)		74 (61.67)	6 (42.86)	

Causes of infertility	Tubal factor	35 (6.78)	17 (6.67)	0.445	188 (73.73)	193 (75.69)	0.780	1127 (78.21)	143 (73.71)	0.146	150 (77.32)	143 (73.71)	0.693	99 (82.50)	12 (85.71)	0.940
	Uterine factor	73 (14.15)	45 (17.65)		21 (8.24)	17 (6.67)		115 (7.98)	14 (7.22)		13 (6.70)	14 (7.22)		9 (7.50)	1 (7.14)	
	Others	236 (45.74)	142 (55.69)		46 (18.04)	45 (17.65)		199 (13.81)	37 (19.07)		31 (15.98)	37 (19.07)		12 (10.00)	1 (7.14)	

BMI (kg/m^2^)		22.98 ± 2.92	22.72 ± 2.81	0.227	22.53 ± 2.74	22.72 ± 2.81	0.452	23.51 ± 2.89	23.69 ± 3.16	0.476	23.54 ± 3.01	23.69 ± 3.16	0.650	23.69 ± 2.93	22.81 ± 3.78	0.302

FSH (IU/L)		6.2 ± 1.55	6.13 ± 1.36	0.555	6.14 ± 1.63	6.13 ± 1.36	0.955	6.69 ± 1.90	6.27 ± 1.37	*p* ≤ 0.01^*∗∗*^	6.38 ± 1.48	6.27 ± 1.37	0.450	7.56 ± 1.95	7.53 ± 2.09	0.955

LH (IU/L)		5.360 (3.9, 7.5)	5.440 (4.0, 6.9)	0.733	5.480 (3.9, 7.7)	5.440 (4.0, 6.9)	0.733	5.58 ± 4.16	5.07 ± 1.96	0.004^*∗∗*^	5.21 ± 2.72	5.07 ± 1.96	0.560	4.770 (3.7, 6.5)	6.010 (2.8, 7.6)	0.856

*E*_2_ (pg/ml)		34.550 (26.5, 45.3)	34.700 (27.1, 45.2)	0.849	35.500 (27.2, 45.1)	34.700 (27.1, 45.2)	0.849	33.600 (26.2, 43.9)	35.065 (26.2, 43.3)	0.885	33.605 (26.8, 43.6)	35.065 (26.2, 43.3)	0.951	38.000 (26.8, 49.9)	35.050 (23.5, 45.3)	0.363

AMH (ng/ml)		5.30 ± 2.77	5.12 ± 2.69	0.372	5.06 ± 2.29	5.12 ± 2.69	0.807	3.63 ± 2.19	3.66 ± 2.27	0.879	3.67 ± 1.99	3.66 ± 2.27	0.938	3.21 ± 2.27	2.92 ± 2.05	0.654

AFC (*n*)		15.75 ± 4.42	17.06 ± 3.56	*p* ≤ 0.01^*∗∗*^	16.94 ± 3.52	17.06 ± 3.56	0.698	13.94 ± 4.37	15.04 ± 4.05	0.001^*∗∗*^	15.55 ± 4.09	15.04 ± 4.05	0.213	12.93 ± 4.41	11.50 ± 3.30	0.241

^*∗*^ *p* < 0.05, ^*∗∗*^*p* < 0.01.

**Table 2:** Comparison between the dual trigger group and the HCG alone group before and after matching: characteristics of patients at stimulation and laboratory results.

	High responders						Normal responders						Poor responders		
	Before matching			After matching			Before matching			After matching					
	HCG alone trigger	Dual trigger	*P*	HCG alone trigger	Dual trigger	*P*	HCG alone trigger	Dual trigger	*P*	HCG alone trigger	Dual trigger	*P*	HCG alone trigger	Dual trigger	*P*
Sample size	*n* = 516	*n* = 255	—	*n* = 255	*n* = 255	—	*n* = 1441	*n* = 194	—	*n* = 194	*n* = 194	—	*n* = 120	*n* = 14	—
Total dose of Gn (IU)	1743.51 ± 700.32	1710.29 ± 651.33	0.526	1699.36 ± 617.88	1710.29 ± 651.33	0.846	1879.21 ± 772.48	1928.74 ± 813.48	0.405	1858.18 ± 768.47	1928.74 ± 813.48	0.380	2060.83 ± 886.78	1930.36 ± 843.54	0.602
Duration of stimulation (d)	9.82 ± 1.73	9.82 ± 1.58	0.977	9.82 ± 1.61	9.82 ± 1.58	0.956	9.54 ± 1.77	9.57 ± 1.89	0.862	9.43 ± 1.78	9.57 ± 1.89	0.456	9.64 ± 2.30	8.57 ± 1.83	0.096
Number of large follicles above 14 mm in diameter on the trigger day (*n*)	13.89 ± 3.92	14.24 ± 3.80	0.232	14.22 ± 3.95	14.24 ± 3.80	0.945	8.52 ± 3.05	9.70 ± 3.28	*p* ≤ 0.01^*∗∗*^	9.68 ± 3.39	9.70 ± 3.28	0.951	4.06 ± 2.00	5.43 ± 3.61	0.185
Serum *E*_2_ on the trigger day (pg/ml)	4797.25 ± 1719.12	5273.92 ± 1843.72	*p* ≤ 0.01^*∗∗*^	5197.81 ± 1790.58	5273.92 ± 1843.72	0.636	2227.37 ± 731.96	2490.73 ± 777.78	*p* ≤ 0.01^*∗∗*^	2453.86 ± 796.47	2490.73 ± 777.78	0.645	1265.29 ± 513.97	1456.04 ± 1011.21	0.498
Serum LH on the trigger day (IU/L)	2.005 (1.3, 3.2)	2.130 (1.4, 3.4)	0.213	2.090 (1.4, 3.1)	2.130 (1.4, 3.4)	0.535	3.19 ± 2.50	2.97 ± 1.89	0.223	2.94 ± 2.30	2.97 ± 1.89	0.910	4.125 (2.6, 5.7)	3.670 (2.0, 5.4)	0.508
Serum progesterone (P) on the trigger day (ng/ml)	0.90 ± 0.50	0.94 ± 0.65	0.332	0.94 ± 0.51	0.94 ± 0.65	0.993	0.560 (0.4, 0.8)	0.585 (0.4, 0.9)	0.289	0.585 (0.4, 0.8)	0.585 (0.4, 0.9)	0.528	0.390 (0.3, 0.6)	0.340 (0.3, 0.5)	0.413
Endometrial thickness on the trigger day (cm)	1.05 ± 0.19	1.06 ± 0.18	0.640	1.05 ± 0.18	1.06 ± 0.18	0.488	1.05 ± 0.19	1.07 ± 0.20	0.137	1.06 ± 0.19	1.07 ± 0.20	0.631	1.03 ± 0.19	1.02 ± 0.17	0.934
The dose of HCG (IU)	6127.91 ± 1763.47	3937.25 ± 2132.42	<0.001^*∗∗*^	5988.24 ± 1728.60	3937.25 ± 2132.42	<0.001^*∗∗*^	8015.96 ± 1193.92	5097.94 ± 1831.13	<0.001^*∗∗*^	7706.19 ± 1447.26	5097.94 ± 1831.13	<0.001^*∗∗*^	8350.00 ± 958.38	6000.00 ± 2183.86	0.001^*∗∗*^
Number of oocytes (*n*)	—	—	—	16.94 ± 5.08	17.53 ± 4.88	0.186	—	—	—	9.89 ± 3.19	10.46 ± 3.04	0.074	2.24 ± 0.86	2.14 ± 1.03	0.691
Oocytes retrieval rate (%)	—	—	—	123 ± 35	129 ± 42	0.102	—	—	—	109 ± 39	114 ± 40	0.199	66 ± 37	58 ± 39	0.448
Fertilization rate. (%)	—	—	—	62 ± 20	60 ± 18	0.179	—	—	—	65 ± 21	68 ± 19	0.107	64 ± 35	55 ± 34	0.392
High-quality embryo rate (%)	—	—	—	54 ± 24	53 ± 26	0.703	—	—	—	56 ± 30	53 ± 31	0.233	63 ± 43	47 ± 48	0.267

^*∗*^ *p* < 0.05, ^*∗∗*^*p* < 0.01.

**Table 3:** Comparison between the dual trigger group and the HCG alone group after matching: characteristics of patients at pregnancy outcomes.

	High responders				Normal responders				Poor responders			
	HCG alone trigger	Dual trigger	*χ* ^2^	*P*	HCG alone trigger	Dual trigger	*χ* ^2^	*P*	HCG alone trigger	Dual trigger	*χ* ^2^	*P*
Sample size	*n* = 255	*n* = 255			*n* = 194	*n* = 194			*n* = 120	*n* = 14		
Embryo transfer rate (%, *n*)	29.80 (76/255)	22.35 (57/255)	3.672	0.055	70.62 (137/194)	67.01 (130/194)	0.588	0.443	52.50 (63/120)	42.86 (6/14)	9.905	0.002^*∗∗*^
Biochemical pregnancy rate (%, *n*)	75.00 (57/76)	68.42 (39/57)	0.702	0.402	67.88 (93/137)	73.08 (95/130)	0.864	0.353	50.79 (32/63)	50.00 (3/6)	<0.001	1.000
Clinical pregnancy rate (%, *n*)	65.79 (50/76)	63.16 (36/57)	0.099	0.753	61.31 (84/137)	64.62 (84/130)	0.312	0.577	44.44 (28/63)	50.00 (3/6)	<0.001	1.000
Live birth rate (%, *n*)	82.00 (41/50)	69.44 (25/36)	1.849	0.174	76.19 (64/84)	82.14 (69/84)	0.902	0.342	82.14 (23/28)	66.67 (2/3)	<0.001	1.000
Abortion rate (%, *n*)	12.00 (6/50)	25.00 (9/36)	2.456	0.117	21.43 (18/84)	16.67 (14/84)	0.618	0.432	17.86 (5/28)	33.33 (1/3)	<0.001	1.000
Transplant cancellation rate due to the prevention of OHSS (%, *n*)	82.68 (148/179)	82.32 (163/198)	0.008	0.927	52.63 (30/57)	42.19 (27/64)	1.320	0.251	0 (0/57)	0 (0/8)	—	—
OHSS incidence rate (%, *n*)	3.92 (10/255)	1.96 (5/255)	<0.001	1.000	0.52 (1/194)	1.03 (2/194)	<0.001	1.000	—	—	—	—

^*∗*^ *p* < 0.05, ^*∗∗*^*p* < 0.01.

## Data Availability

The data that support the findings of this study are available from the Hospital for Reproductive Medicine Affiliated to Shandong University, but restrictions apply to the availability of these data, which were used under license for the current study and so are not publicly available. Data are, however, available from the correspondence authors upon reasonable request.
